# Association of Sarcopenia with Lower Adiponectin Levels and Reduced Estimated Appendicular Lean Mass in Patients with Metabolic Syndrome: A Cross-Sectional Study

**DOI:** 10.3390/diseases14020072

**Published:** 2026-02-14

**Authors:** Juan Antonio Suárez-Cuenca, Pablo Zermeño-Ugalde, Diana Elisa Díaz-Jiménez, Juan Antonio Pineda-Juárez, Deyanhira Palacios-Colunga, Alejandro Hernández-Patricio, Eduardo Vera-Gómez, Areli Romero-López, María Fernanda Kuri-Pineda, Andrea Ramírez-Coyotecatl, Dulce Cecilia Vázquez-Ramos, José Gutiérrez-Salinas, Silvia García, Christian Alejandro Delaflor-Wagner, Christian Gabriel Toledo-Lozano, Luis Montiel-López, María Angélica Díaz-Aranda, Alberto Melchor-López

**Affiliations:** 1 Laboratorio de Metabolismo Experimental e Investigación Clínica, CMN “20 de Noviembre”, ISSSTE, San Lorenzo 502, Colonia del Valle Sur, Benito Juárez, Mexico City 03104, Mexico; juan.pineda@issste.gob.mx (J.A.P.-J.); patricio.alejandro.issste@gmail.com (A.H.-P.); eduardo.vera.issste.20nov@gmail.com (E.V.-G.); areromlo@gmail.com (A.R.-L.); ferkurimet.exp5.issste@gmail.com (M.F.K.-P.); mc21raca2684@facmed.unam.mx (A.R.-C.); dulce.vazquez.ramos@estudiante.uacm.edu.mx (D.C.V.-R.); christian.toledo@issste.gob.mx (C.G.T.-L.); yelidiazaranda@gmail.com (M.A.D.-A.); 2 Facultad de Enfermería y Nutrición, Universidad Autónoma de San Luis Potosí, San Luis Potosí 78240, Mexico; pablo.zermeno@uaslp.mx (P.Z.-U.); deyanhira.palacios@uaslp.mx (D.P.-C.); 3 Hospital General de Zona No. 8 y UMF “Dr. Gilberto Flores Izquierdo”, Instituto Mexicano del Seguro Social, Rio Magdalena 289, Col. Tizapan San Ángel, Alcaldía Álvaro Obregón, Mexico City 01090, Mexico; diana_dj1@hotmail.com (D.E.D.-J.); dralbertomelchor@gmail.com (A.M.-L.); 4 Laboratorio de Bioquímica y Medicina Experimental, CMN “20 de Noviembre”, ISSSTE, San Lorenzo 502, Colonia del Valle Sur, Benito Juárez, Mexico City 03104, Mexico; jose.gutierrezsa@issste.gob.mx; 5 Servicio de Investigación Clínica, CMN “20 de Noviembre”, ISSSTE, San Lorenzo 502, Colonia del Valle Sur, Benito Juárez, Mexico City 03104, Mexico; rolasil@yahoo.com.mx (S.G.); christian.delaflor@issste.gob.mx (C.A.D.-W.); 6 Servicio de Medicina Interna, CMN “20 de Noviembre”, ISSSTE, Félix Cuevas 540, Colonia del Valle Sur, Benito Juárez, Mexico City 03104, Mexico; luis.montiel@issste.gob.mx

**Keywords:** sarcopenia, estimated appendicular lean mass, body composition, adiponectin, metabolic syndrome

## Abstract

**Background:** Sarcopenia is a progressive muscle disorder associated with metabolic syndrome (MS), in which early impairments in muscle strength and quality precede muscle mass loss. Simple, non-invasive measures such as handgrip strength, estimated appendicular skeletal muscle mass (eASM), and phase angle (PA) may aid early detection, while adipokines link muscle dysfunction to metabolic regulation. **Objective:** In the present study, we aimed to evaluate the association between sarcopenia markers and PA in patients with MS. **Methods:** A cross-sectional study was conducted in patients with MS, at a third-level hospital in Mexico City. Sarcopenia was assessed by handgrip strength and eASM; body composition and PA were measured using bioelectrical impedance; and plasma adipokines were quantified by ELISA. **Results:** Seventy-four (mean age, 57.7 years; 75% female; BMI, 32.5 kg/m^2^) participants with MS were included. Handgrip strength correlated with eASM (r = 0.64; *p* < 0.01) and PA (rho = 0.43; *p* < 0.01), and eASM also correlated with PA (rho = 0.40; *p* < 0.01) and predicted higher PA values (OR = 2.74; *p* = 0.042). The sarcopenic subgroup had lower brachial circumference and plasma adiponectin. **Conclusions:** Sarcopenia is frequent in MS and associated with lower adiponectin, suggesting a vulnerable condition. Functional/structural markers of sarcopenia showed significant correlation with PA, whereas combined methods may enhance the early detection and management of muscle deterioration in metabolic disease.

## 1. Introduction

Sarcopenia derives from Greek and means scarcity (*penia*) of flesh (*sarx*). This concept refers exclusively to skeletal muscle tissue and does not include other types such as smooth, cardiac, or myoepithelial muscle tissue [1]. Sarcopenia is an age-related disease that mainly involves decreasing muscle mass, strength, and function, affecting the nutritional status of individuals [2]. The coexistence of sarcopenia and metabolic syndrome (MS), often referred to as metabolic sarcopenia or sarcopenic obesity, is increasingly recognized, with epidemiological evidence showing a significantly higher prevalence of sarcopenia among individuals with MS, reaching approximately 11% in the general MS population and up to 16% in hospitalized patients, depending on diagnostic criteria [3].

Accurate assessment of muscle mass and function is therefore essential for sarcopenia identification, and measures such as handgrip strength and appendicular skeletal muscle mass demonstrate strong correlations with imaging and body composition techniques, including magnetic resonance imaging (MRI), dual-energy X-ray absorptiometry (DXA), and bioelectrical impedance analysis (BIA). Although these techniques are considered reference standards for evaluating skeletal muscle mass and quality, their high cost, limited accessibility, and prolonged acquisition time limit their routine clinical and large-scale application, highlighting the need for simpler, non-invasive tools for early detection and monitoring of muscle alterations. Currently, low muscle strength is regarded as the primary criterion for sarcopenia identification and is most commonly assessed using handgrip strength, a validated and reliable measure widely applied in both clinical practice and research [4].

Beyond the loss of muscle mass and strength, growing evidence suggests that adipose tissue plays a relevant role in sarcopenia development and progression. This tissue is an endocrine organ with high metabolic activity, and it secretes hormones known as adipokines, such as adiponectin, leptin, resistin, interleukin 6 (IL-6), and tumor necrosis factor-α (TNF-α). Recent research indicates that leptin, adiponectin, and resistin exert regulatory effects on skeletal muscle [2]. On the other hand, to maintain homeostasis, the effect of adiponectin must be considered. Adiponectin is an adipokine derived from white adipose tissue with anti-inflammatory activity, and it binds to adiponectin receptor 1 (AdipoR1), expressed in skeletal muscle, and adiponectin receptor 2 (AdipoR2), expressed in the liver. In muscle, it inhibits proteolysis, enhances myogenesis, increases insulin sensitivity, and inhibits inflammation in myotubes [2].

Excessive accumulation of adipose tissue leads to lipid deposition in various tissues, including muscle tissue. This intramuscular fat accumulation triggers an inflammatory response through the production of reactive oxygen species and increased C-reactive protein, resulting in mitochondrial damage within muscle tissue and consequently reduced muscle function [5]. Other pro-inflammatory cytokines released as a result of adipose tissue accumulation, such as IL-6 and TNF-α, act as signaling molecules that recruit inflammatory cells into the muscle, triggering a vicious cycle that maintains the muscle in a chronic inflammatory state. This state induces apoptosis of myocytes and reduces the muscle’s regenerative capacity, which has been observed in patients with sarcopenia [6].

The presence of sarcopenia in individuals with MS has important clinical and metabolic implications and is associated with increased cardiovascular risk, physical disability, and all-cause mortality. This bidirectional interaction between muscle dysfunction and metabolic inflammation establishes a vicious cycle that accelerates functional and metabolic deterioration [4]. Early detection is therefore essential, as declines in muscle strength and quality often precede muscle mass loss, enabling timely nutritional, exercise, and metabolic interventions to preserve muscle function, improve insulin sensitivity, and reduce cardiometabolic risk.

Simple, non-invasive, and easily applicable tools, such as handgrip strength and phase angle (PA), are particularly useful for the early detection of muscle alterations in populations with MS, in whom access to imaging techniques may be limited [7,8]. Consequently, early identification of sarcopenia represents a key strategy for improving prognosis and quality of life in these patients. In the present study, we aimed to evaluate the clinical utility of these non-invasive markers as a complementary measure for assessing sarcopenia and muscle health in the context of cardiometabolic risk, like patients with MS.

## 2. Methods

A cross-sectional study was conducted at Centro Médico Nacional 20 de Noviembre, ISSSTE, Mexico City, Mexico, between 2018 and 2023.

Study population. Patients were diagnosed with MS according to the NCEP-ATP III classification [9]. Exclusion criteria included chronic renal failure (KDIGO stage ≥ 3), cancer, HIV, chronic heart failure, chronic liver disease, and COPD. The study was approved by the Joint Committee of Bioethics, Biosafety and Research of the Centro Médico Nacional 20 de Noviembre (ID 083.2018), and all participants provided written informed consent.

**Data Collection**. All patients underwent an initial interview to obtain personal data, comorbidities, and anthropometric measurements. 

**Anthropometry.** Anthropometric parameters included weight, height, and BMI (Body Mass Index), as well as neck, brachial, waist, and hip circumferences. Patients were weighed with a calibrated scale, SECA^®^ scale (SECA^®^, Chino, CA, USA) (model 813; capacity 200 kg; accuracy ±100 g), and were asked to remove all extra weight to avoid bias. Height was measured with an SECA^®^ model 220 wall stadimeter with a capacity of 230 cm and an accuracy of ±1 mm, and likewise, the average circumference of the neck, arm, waist, and hip was measured with SECA metric tape (cm).

**Sarcopenia assessment.** Sarcopenia was assessed by two methods: handgrip strength and estimated appendicular skeletal muscle mass (eASM). Briefly, handgrip strength was measured using a Takei^®^ hand dynamometer (Takei Scientific Instruments Co., Ltd, Tokyo, Japan) with a measurement range of 0 to 100 kg. Patients carried out 3 consecutive measurements with the dominant hand, and the mean value of the handgrip strength tries was calculated. Sarcopenia was diagnosed according to the guidelines of the European Working Group on Sarcopenia in Older People (EWGSOP2), based on handgrip strength, using cutoff values of <27 kg for men and <16 kg for women [4]. On the other hand, eASM was estimated using anthropometric predictive equations based on body weight, height, age, and sex, according to Lee et al.’s equation [10]: eASM (kg) = 0.244 × Weight (kg) + 7.8 × Height (m) + 6.6 × Sex(1 for men, 0 for women) − 0.098 × Age (years) + Race (0 for White and Hispanic; 1.9 for Black; −1.6 for Asian) − 3.3.

**Body composition.** Body composition was assessed by multi-frequency bioelectrical impedance analysis using a Bodystat QuadScan 4000 device, obtaining phase angle (PA), calculated at 50 kHz, and additional parameters related to cellular integrity and body composition. Measurements were performed under standardized conditions, including a 1-h fasting period, avoidance of vigorous physical activity and alcohol consumption for 24 h prior to testing, and removal of metallic objects. Participants were evaluated in the supine position using a four-electrode configuration, with two electrodes placed on the right hand and two on the right foot.

**Plasma Biochemical Tests.** Blood samples were obtained by puncture in the peripheral vein during the initial interview, and further processed through routine automatized laboratory methods. Biochemical data such as lipid profile (total cholesterol, LDL cholesterol, HDL cholesterol, triglycerides) and plasma fasting glucose and data were collected from the digital clinical records.

**Plasma Cytokines.** Cytokines and biomarkers were quantified using commercial ELISA kits: IL-1β (cat. BMS224-2TEN), IL-10 (cat. BMS215-2TEN), and adiponectin (cat. BMS2032-2; Thermo Fisher Scientific, Waltham, MA, USA), as well as sarcolipin (SLN; cat. 027956; US Biological Life Sciences, Salem, MA, USA), following manufacturers’ instructions and analyzed with an ELISA plate reader.

**Statistical Analysis.** Statistical analyses were performed using jamovi 2.6.26 software. The normality of the distribution was assessed using the Shapiro–Wilk or Kolmogorov–Smirnov test, and depending on this distribution, the results are presented as the mean ± standard deviation or P50 (P25, P75) for continuous variables. Likewise, categorical variables are presented as n (%). Inferential analyses were performed either through Student’s T test or the Mann–Whitney U test, according to normality or number of groups. Correlation analyses and Odds Ratio (OR) were also performed. For exploratory purposes, phase angle (PA) and estimated appendicular skeletal muscle mass (eASM) were additionally dichotomized using sample-derived cutoffs (median values). These thresholds were not intended as diagnostic criteria, but rather to explore potential associations within this specific population. Statistical significance was considered if *p* ˂ 0.05.

## 3. Results

The study population comprised 74 patients with MS, with a mean age of 57.7 years, 75.7% being female, mean Body Mass Index of 32.5 kg/m^2^, and metabolic risk mainly denoted by a waist circumference of 104 ± 13.1 cm and mean plasma glucose of 119 mg/dL, without significant lipid disorders (Table 1 and Table 2). 

**Figure 1:**
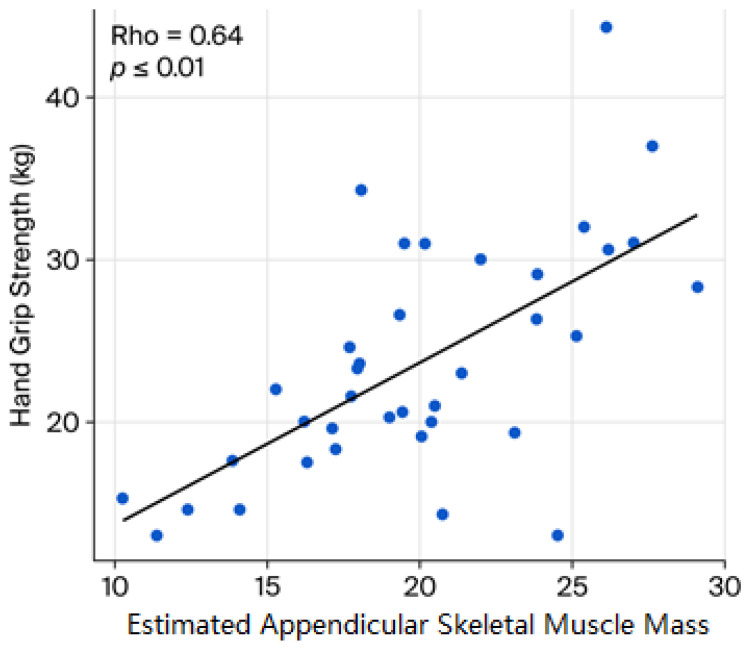
Relationship between sarcopenia markers. The graphic shows the correlation between the estimated appendicular skeletal muscle mass (eASM) and the handgrip strength. Spearman’s rank correlation coefficient was applied.

The presence of sarcopenia was defined by the handgrip strength, which significantly correlated with the eASM (Pearson rho, 0.64; *p* ≤ 0.01; Figure 1), an additional marker of sarcopenia. The subpopulation with sarcopenia was characterized by older age and lower brachial circumference, as well as lower plasma adiponectin.

The PA showed a median value of 5.6° (IQR: 5.3–6.4). When stratified by sarcopenia status, patients with sarcopenia showed a PA of 5.5° (4.9–6.0), whereas those without sarcopenia had median values of 5.6° (5.5–6.5), without statistically significant differences.

**Figure 2:**
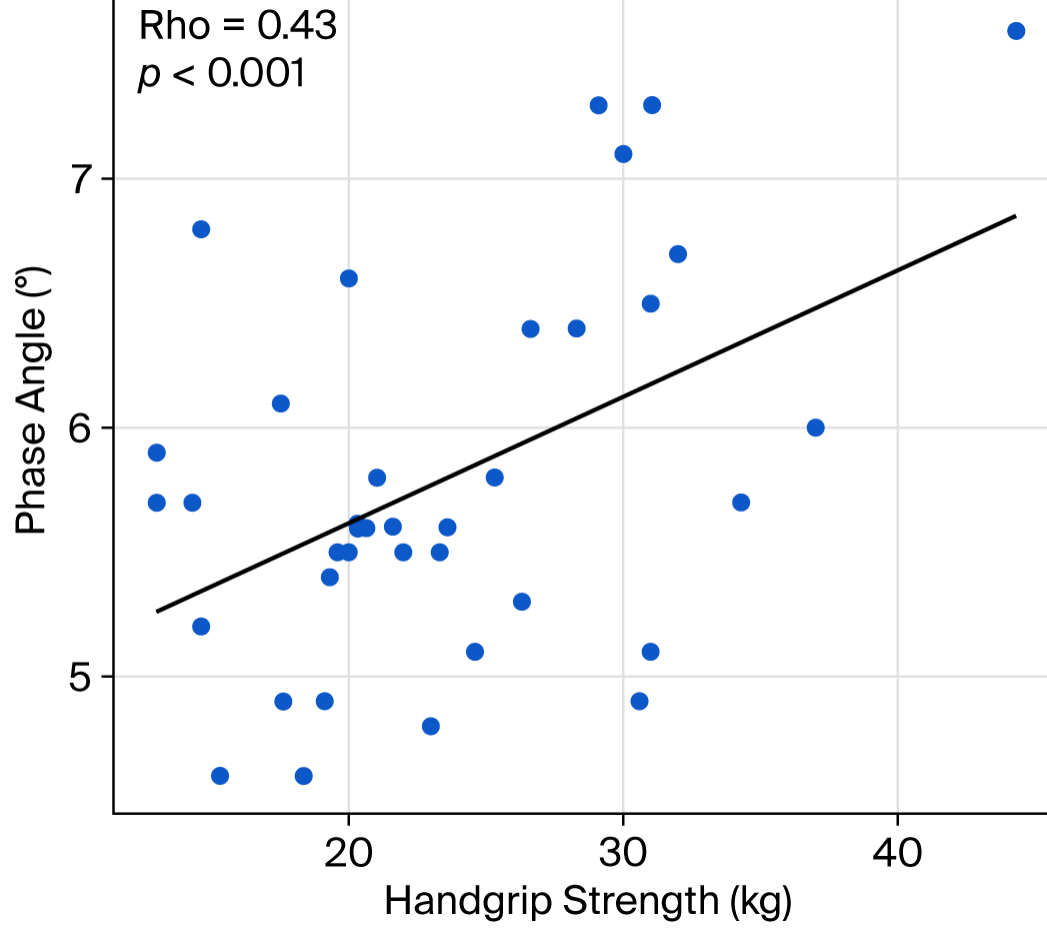
Sarcopenia markers and phase angle–handgrip strength. The graphic shows the correlation between handgrip strength and the phase angle. Spearman’s rank correlation coefficient was applied.

**Figure 3:**
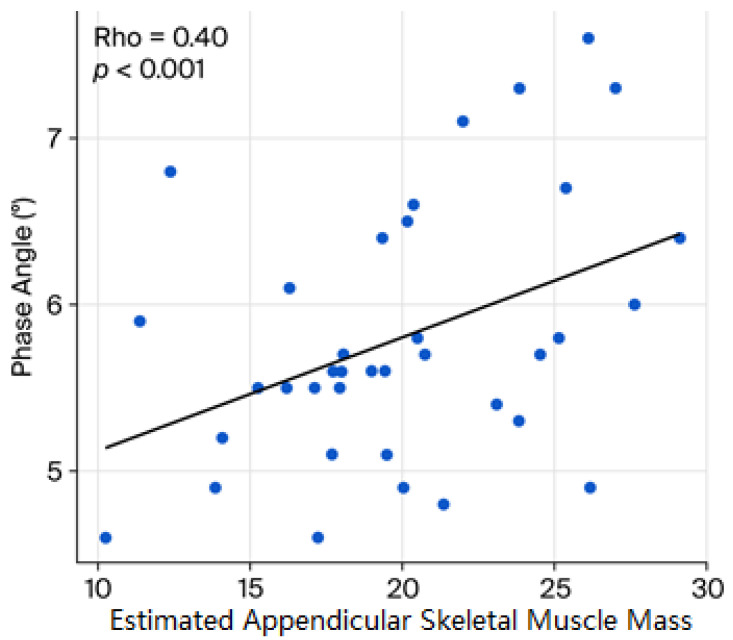
Sarcopenia markers and phase angle. The graphic shows the correlation between the estimated appendicular skeletal muscle mass (eASM) and the phase angle. Spearman’s rank correlation coefficient was applied.

**Figure 4:**
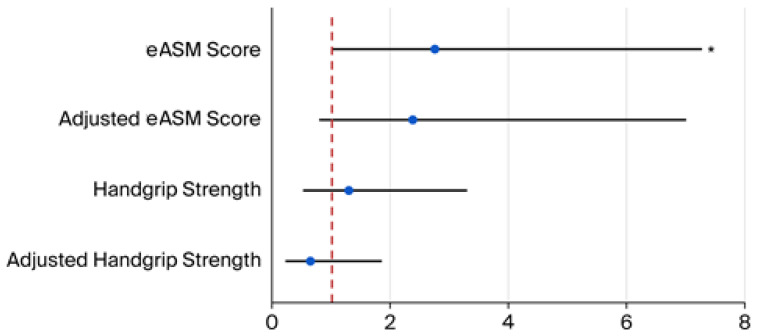
Association between sarcopenia markers and phase angle. Forest plot shows the risk of sarcopenia markers to predict higher phase angles (considered as a median cutoff of 5.6°). eASM (cutoff, 19.5): OR, 2.74; CI95%, 1.0 to 7.3; *p* = 0.042. Adjusted eASM (cutoff, 19.5): OR, 2.4; CI95%, 0.8 to 7.0; *p* = 0.12. Handgrip strength (cutoff, 22 kg): OR, 1.3; CI95%, 0.5 to 3.3; *p* = 0.28. Adjusted handgrip strength (cutoff, 22 kg): OR, 0.62; CI95%, 0.2 to 1.8; *p* = 0.39. OR adjustments were performed by age and sex. (*) = *p* < 0.05. Abbreviations: estimated appendicular skeletal muscle mass (eASM).

Finally, sarcopenia, either reflected by handgrip strength or the eASM, showed a significant and positive correlation with values of PA (handgrip strength ρ = 0.43, *p* ˂ 0.01; eASM ρ = 0.40, *p* ˂ 0.01, respectively; Figure 2 and Figure 3). In exploratory analyses, higher eASM values, using a sample-derived cutoff (19.5 kg), were associated with higher PA values (OR 2.74, CI95% 1.0–7.3, *p* = 0.042; Figure 4).

## 4. Discussion

In this study of patients with MS, the high prevalence of obesity and cardiometabolic risk, as indicated by waist circumference and elevated fasting plasma glucose, is consistent with previous reports in middle-aged, predominantly female populations [11]. Interestingly, significant lipid abnormalities were not observed, which may reflect the clinical heterogeneity of MS or the influence of pharmacological treatment [12]. 

The presence of sarcopenia was identified by two complementary approaches: handgrip strength and eASM; both markers showed a significant positive correlation. Notably, handgrip strength exhibited the highest effect size (*p* < 0.01, rho = 0.64), underscoring its value as a functional marker. Other sarcopenia markers, like eASM as well as anthropometric surrogates, yielded comparable insights. Chattopadhyay et al. reported the usefulness of mid-arm circumference (*p* < 0.006, rho = 0.37) and hand length (*p* < 0.022, rho = 0.32) [13]. Prior evidence has validated these measures as sensitive diagnostic tools for sarcopenia, either in healthy populations (*p* = 0.002, rho = 0.029) or across several disease conditions (*p* < 0.54, rho = 0.001) [14,15], particularly in individuals with obesity and metabolic comorbidities, and in geriatric and post-menopausal women over 50 years (*p* < 0.006, rho = 0.267) [4,13,14,15,16,17,18,19]. Collectively, these findings suggest that eASM or its anthropometric equivalents maintain a significant relationship with muscle function, as measured by handgrip strength.

The main finding of the present study is that the subgroup with sarcopenia was characterized by lower brachial circumference and reduced plasma adiponectin, suggesting a more vulnerable phenotype. This is of relevance since adiponectin has been shown to directly influence skeletal muscle integrity. For example, in muscle cells, adiponectin signaling promotes myogenesis and inhibits proteolysis via the IRS-1/Akt pathway, thereby counteracting atrophic processes [3]. Likewise, adiponectin exerts anti-inflammatory effects and plays a protective role in muscle and metabolic homeostasis [20]. Adiponectin modulates systemic and local inflammation: it suppresses pro-inflammatory cytokines such as TNF-α and IL-6, while up-regulating anti-inflammatory mediators (IL-10, IL-1 receptor antagonist) in monocytes/macrophages [20]. Since chronic low-grade inflammation is a known driver of muscle catabolism and sarcopenia, lower adiponectin may remove an important brake on this process, aligning with the more vulnerable phenotype observed in the subgroup with sarcopenia. Moreover, the concurrency of low adiponectin with reduced brachial circumference may reflect a dual hit: less protective adipokine signaling and less muscle mass to utilize/adapt. This “double vulnerability” may predispose to worse functional outcomes, particularly important in the context of MS and obesity. In this regard, inverse associations between adiponectin and skeletal muscle mass indices have been reported, which suggests that the interpretation of adiponectin levels in sarcopenia may depend on the specific metabolic/phenotypic context, for example, sarcopenic obesity vs. “pure” sarcopenia [21].

Another relevant finding of the present study is that sarcopenia markers were further associated with PA values. PA, derived from bioelectrical impedance analysis, is increasingly recognized as a non-invasive biomarker of cellular integrity and body composition, with strong prognostic implications in diverse clinical settings [7]. In the present study, the PA values are comparable to those reported in patients with MS, with a typical range between approximately 5.0 and 5.8 [22]. Furthermore, the PA values in the present study were significantly related to handgrip strength values (*p* < 0.001, rho = 0.43), which is in line with findings by Özge et al., who reported significant associations between PA and handgrip strength (*p* < 0.001, rho = 0.523) and eASM (*p* < 0.001, rho = 0.335) in 135 institutionalized older adults [16]. Similarly, Yamanaka et al. observed a pronounced link between PA and handgrip strength among hospitalized patients with head and neck cancer (*p* < 0.001, rho = 0.649) [17].

The positive correlations of handgrip strength and eASM with PA reinforce the role of the latter in evaluating functional and nutritional status in patients with MS and further support the concept that loss of muscle mass and function directly compromises cellular quality, as also reported in sarcopenic obesity and metabolic disease populations [23,24]. We further validated the results obtained, through Benjamini–Hochberg false discovery rate (FDR) correction, where all examined correlations remained statistically significant. Specifically, the association between eASM and handgrip strength showed an adjusted *p* value of 0.01 (FDR-adjusted *p* = 0.01). The correlation between handgrip strength and PA also remained significant (*p* = 0.001; FDR-adjusted *p* = 0.003). Likewise, the association between eASM and PA persisted after correction (*p* = 0.001; FDR-adjusted *p* = 0.0015).

In our study, we highlight the importance of integrating anthropometric measurements, handgrip strength, and body composition parameters to enable effective muscle screening without relying on costly or limited-access techniques. In line with these findings, the study by Luo, S. et al. [25] supports the use of body circumferences combined with basic body composition indicators as the most commonly applied and clinically useful parameters for assessing muscle status. This integrated approach facilitates the early detection of muscle alterations in clinical settings, supporting timely and targeted interventions [26]. Furthermore, several studies have demonstrated that these anthropometric measurements and functional indicators show a strong agreement with dual-energy X-ray absorptiometry (DEXA), which is considered a reference method for assessing skeletal muscle mass [27].

## 5. Conclusions

Taken together, these findings highlight the need for systematic evaluation of sarcopenia in MS patients, even in relatively early stages where obesity may mask muscle deterioration. The combined use of functional (handgrip strength), estimated appendicular skeletal muscle mass (eASM), and phase angle assessments provides a comprehensive approach to characterize musculoskeletal status and metabolic risk. These results underscore the clinical relevance of integrating sarcopenia screening into MS management and support early intervention strategies aimed at improving muscle function and cardiometabolic outcomes.

Study Limitations. The small sample size may limit the generalizability of our findings, and the cross-sectional design precludes establishing causal relationships between variables. The use of the Lee equation to estimate eASM was originally validated in specific ethnic populations, and its application in Mexican adults may introduce misclassification bias. Additionally, the equation incorporates weight, height, age, and sex, which may lead to collinearity when eASM estimates are analyzed alongside related variables such as BMI or other anthropometric indicators, potentially affecting the robustness of the statistical models. Finally, the use of sample-derived cutoffs for phase angle and eASM may limit external validity and should be considered exploratory.

## Figures and Tables

**Table 1:** Study population characteristics (*n* = 74).

	All (*n* = 74)	Sarcopenia * (*n* = 27)	w/o Sarcopenia * (*n* = 47)	*p* Value
Age (y-o)	54.7 ± 9.3	57.9 ± 7.1	52.8 ± 10.1	0.02
Female gender n (%)	56 (75.7)	22 (81.5)	34 (72.3)	0.30
PFG (mg/dL)	119.5 (98.7, 138.2)	127.5 (106.2, 138.2)	112.5 (98.7, 139.7)	0.49
Total cholesterol (mg/dL)	161.2 ± 48.7	182.0 ± 47.2	151.0 ± 47.1	0.09
HDLc (mg/dL)	42.2 (35.8–51.1)	48.1 (44–52)	41.9 (33–45.6)	0.09
LDLc (mg/dL)	80.4 (71.0–123.1)	107.7 (66.3–127.7)	79.8 (74.0, 122.5)	0.84
Triglycerides (mg/dL)	125.0 (106.2–140.7)	122.5 (106–136.2)	127.0 (109.0–167.5)	0.40
Albumin (g/dL)	4.3 (4.1–4.4)	4.4 (4.2–4.4)	4.3 (4.1–4.3)	0.34

Categorical variables are presented as frequencies and percentages and were compared using the chi-square test. Normality of continuous variables was assessed using the Kolmogorov–Smirnov test. Normally distributed variables are expressed as the mean ± SD and compared using the unpaired Student’s *t*-test, whereas non-normally distributed variables are presented as the median (interquartile range) and compared using the Mann–Whitney U test. Abbreviations: PFG, plasma fasting glucose; HDLc and LDLc: high- and low-density lipoprotein cholesterol, respectively. (*) Sarcopenia was defined according to handgrip strength test.

**Table 2:** Anthropometric measures and pro-inflammatory mediators.

	All (*n* = 74)	Sarcopenia (*n* = 27)	w/o Sarcopenia (*n* = 47)	*p* Value
Handgrip strength (kg)	23.5 ± 7.1	17.5 ± 4.2	27 ± 6.1	<0.001
Phase Angle (°)	5.6 (5.3–6.4)	5.5 (4.9–6)	5.6 (5.5–6.5)	0.09
eASM	19.9 ± 4.6	17.2 ± 4.4	21.5 ± 3.9	<0.001
Body Mass Index (kg/m^2^)	32.3 ± 5.5	32.1 ± 6.3	32.4 ± 5.0	0.80
Neck circumference (cm)	36.5 (35.3–39.6)	37.0 (34.5–41.0)	36.5 (35.7–39.0)	0.6
Brachial circumference (cm)	34.1 ± 3.9	32.7 ± 4.3	34.9 ± 3.4	0.02
Waist circumference (cm)	104 ± 13.1	103.7 ± 15.4	104.2 ± 11.7	0.87
Hip circumference (cm)	110.9 ± 14	110 ± 13.3	111.3 ± 14.5	0.70
Waist–Hip Ratio	0.9 ± 0.07	0.9 ± 0.07	0.9 ± 0.07	0.96
Waist-to-Height Ratio	65.5 ± 7.6	66.1 ± 9.3	65.2 ± 6.6	0.61
Cytokines				
IL-10 (pg/mL)	33.2 ± 17.5	37.2 ± 14.6	31.3 ± 14.7	0.20
Adiponectin (μg/mL)	12.4 (10.2–20.3)	10.43 (9.69–13.9)	16.1 (11.1–22.1)	0.01
IL-1β1 (pg/mL)	5.6 (4.7–7.0)	5.8 (5.0–6.8)	5.4 (4.6–7.0)	0.60
Sarcolipin (pg/mL)	0.4 ± 0.2	0.4 ± 0.3	0.4 ± 0.1	0.30

Categorical variables are presented as frequencies and percentages and were compared using the chi-square test. Normality of continuous variables was assessed using the Kolmogorov–Smirnov test. Normally distributed variables are expressed as the mean ± SD and compared using the unpaired Student’s *t*-test, whereas non-normally distributed variables are presented as the median (interquartile range) and compared using the Mann–Whitney U test. Abbreviations: eASM, estimated appendicular skeletal muscle mass; IL-10, interleukin 10; IL-1β1, interleukin 1 beta 1.

## Data Availability

The datasets are not publicly available due to privacy policies of the hospital and patient’s sensitive information, but are available from the corresponding author on reasonable request.

## Abbreviations

MS	Metabolic Syndrome
eASM	Appendicular Skeletal Muscle Mass
PA	Phase Angle
